# For the Love of Alma, Black Hopes Matter

**DOI:** 10.1089/pmr.2020.0018

**Published:** 2021-04-21

**Authors:** John Stonestreet

**Affiliations:** HopeCare, Saint Petersburg, Florida, USA.

Breaking bad news to grieving families is never easy. A hospital ethics director once shared with me a time that a wife passed out on the spot upon hearing about her husband's impending “Do Not Resuscitate” order. What kind of long-term toll can traumatic conversations take on grieving families?

In one of my most memorable encounters as a spiritual care provider, I vividly recall reporting to the bedside of Alma, an African American woman suffering from intense pain that the medical team was struggling to diagnose and treat.

When I sat down at Alma's bedside and listened to her story, one of the root causes of her total suffering became crystal clear. Alma was suffering from a broken heart, grieving the recent loss of her husband, whom she had married as a teenager, and with whom she had spent the entirety of her adult life. In addition, Alma was suffering from the emotionally crushing experience of being, in her words (as you will hear in her gripping audio account), “tortured about hospice.”^1^ She was then further disenfranchised by clinicians who, absent Alma's account, might otherwise be lauded for honoring their “duty to the patient,” all in the name of bioethical imperatives and “patient-centered care.”

As Alma's husband languished in the intensive care unit (ICU), teetering on the edge of death, the medical team had quite rationally concluded that the time had come to transition her husband from his current ICU bed to the inpatient hospice unit down the hall. When this suggestion was communicated to Alma, she expressed her fears and misgivings. She understood the prognosis, but she still hoped for a miracle. Ultimately, she was invited to a family meeting to discuss the goals of care.

Going into the meeting, Alma's goal was to make sure that her husband's wishes were honored and that he had fair opportunity to express his goals without duress. Alma said that if hospice is “what he wanted, then by all means he should have it…I just wanted to be sure that there was no coercion. That's all.” As you will hear^1^ so painfully expressed, Alma arrived promptly at the appointed time only to discover that the meeting had already occurred, that her husband was already transitioned to hospice, and that she was not considered an important party to discussions about his care.

Agonizing, suffering the simultaneous loss of her husband, and betrayal by the medical team, Alma languished in grief to the point of serious physical illness requiring hospitalization. Even after Alma was treated in the hospital and improved enough to be discharged, her health continued to spiral downward. Alma's decline was not merely one characterized by excruciating physical pain; rather, the decline affected all aspects of Alma's life: physical, mental, and emotional. Her suffering paralyzed her personal life, it crippled her functioning, and it decimated her productivity. As a result, she fell behind on payments that threatened her granddaughter's ability to continue nursing school. Alma spent entire nights down on her knees, crying out to God, begging for a way out of the deep hole in which she found herself.

Reflecting on Alma's story from the point of our initial encounter in the hospital, it seems to me that there was a critical tipping point that complicated her grief enough to push her over the top: the ICU medical team unwittingly weaponized bioethics to suit their own notions of tidiness and gravely harmed Alma in the process. My goal is not to demonize those clinicians, but to point out how bankrupt bioethics can become if it is not pursued through a lens of person-centered care. This medical team was operating on solid ethical ground, honoring the principles of systemic justice and patient autonomy. But these clinicians prioritized disembodied principles over Alma's embodied personhood.

What I learned from Alma was that communication failures by the medical team, when combined with psychosomatic effects of grief, can complicate grief, and have complicated grief, to the point of ravaging a family member's physical health. Communication failures can be a contributing factor to physical illness, constituting a tangible harm and a violation of the Hippocratic oath. In addition to the ethical costs, these harms can also come at a significant financial cost both to patients and, of course, also to the health care system (complicating shortsighted bioethical calculations of justice). In short, communication failures can turn a family member into a patient (one who might be unlikely to trust any medical caregiver).

Alma nearly “lost her will” to live as a result of complicated grief.^1^ Her troubling experience raises awareness of a more widespread reality. Like the overwhelming majority of African Americans,^3–9^ Alma had a religious and ethnocultural belief in miracles through divine intervention. Such beliefs statistically modify prognosis interpretation,^3,6^ even in the face of physician determinations that further medical intervention would be nonbeneficial. Alma's husband's medical team failed to honor their duty of cultural sensitivity to Alma's beliefs.

Sadly, Alma's case is not as isolated as we might hope. I have personally had the displeasure of sitting and watching an advanced palliative practitioner at a leading academic medical center shame, chide, belittle, and berate an African American family for their ethnocultural beliefs and related hopes. And all this occurred under normal nonpandemic circumstances. But even those many physicians who think that they could never bring themselves to such cruelty still routinely flounder when African Americans express hope for an unlikely miracle. The therapeutic encounter crumbles.^2^

How might Alma's story have turned out differently if the team had been trained in ethnocultural sensitivity for end-of-life transition conversations?^10^ And what if they had begun a family meeting by recognizing and honoring not just the doctors' perspective,^11^ or the nurses' perspective,^12^ but also Alma's hopes?^1^ What emotional impact might it have made if the team had gathered together with Alma around the bedside of her husband, and a physician had placed a supportive hand on her shoulder, as a chaplain offered a prayer for the miracle she hoped for?^2^

For the love of Alma,^1^ and other health care disenfranchisement victims,^13^ black hopes matter.^14^

Until recently, bioethical notions of justice were comparatively hypothetical. But for months now, physician communication trainers have been circulating conversation guides for informing families through telemedicine that, due to current measures, their loved one's hospital ventilator will be removed, hastening death (and current emergency protocols prevent them from having any say in the matter).^15^ Might there also be a need for targeted presentations,^16^ and trainings,^10^ enriching such conversation guides with approaches integrating needed spiritual interventions?^17,*^

The LA Times paints a frightening picture of the potential for COVID-19 to necessitate a raid on *occupied* ventilators.^18^ Families of comatose patients could be forced to give up on their miracle and “pull the plug on Grandma” in favor of a COVID-19 patient with a higher likelihood of survival. Whether this tortured choice becomes necessary in the current wave, or a future wave, of this or another pandemic, it raises three important questions: how might physicians break this news to families? And how might families respond? Most critically, how will physicians acknowledge the family's response?

For the love of Alma, and in solidarity with the suffering that brought her to the very precipice of having “lost her will” to live, act now to stop unnecessary suffering from [unintentional] ethnocultural discrimination: book a specialist to deliver medical grand rounds at your hospital.^2,10,16,17^

In this historic season of peaceful protest for justice, please join this conversation, share your health care experiences of discrimination, and help promote social awareness of the need for a Black Lives Matter for Healthcare website,^19^reminding everyone why/how black hopes matter. By working together in solidarity with suffering patients and families, we can demand and achieve accountability.^21,22^

## Abbreviations Used

ICU: intensive care unit

## Notes

^*^ “While the Joint Commission already requires staff education concerning the unique needs, including those that relate to patient religion and spirituality, of patients at the end of life, the RSCC study indicates that this training requirement is seldom fulfilled. If training were provided it would significantly increase frequency of spiritual care in life-threatening illness.”^9^
